# In-hospital safety outcomes of left atrial appendage occlusion in octogenarians and nonagenarians

**DOI:** 10.1093/europace/euae055

**Published:** 2024-02-23

**Authors:** Mahmoud Ismayl, Hasaan Ahmed, Andrew M Goldsweig, James V Freeman, Mohamad Alkhouli

**Affiliations:** Department of Cardiovascular Medicine, Mayo Clinic, 200 First Street SW, Rochester, MN 55905, USA; Department of Internal Medicine, Creighton University School of Medicine, Omaha, NE, USA; Department of Cardiovascular Medicine, Baystate Medical Center, Springfield, MA, USA; Department of Cardiovascular Medicine, Yale University School of Medicine, New Haven, CT, USA; Department of Cardiovascular Medicine, Mayo Clinic, 200 First Street SW, Rochester, MN 55905, USA

**Keywords:** Atrial fibrillation, LAAO, Octogenarians, Nonagenarians, Outcomes

## Abstract

**Aims:**

Data on safety outcomes of left atrial appendage occlusion (LAAO) in elderly patients are limited. This study aimed to compare the outcomes of LAAO between octogenarians (age 80–89) and nonagenarians (age ≥90) vs. younger patients (age ≤79).

**Methods and results:**

We conducted a retrospective cohort study using the National Inpatient Sample database to identify patients hospitalized for LAAO from 2016 to 2020 and to compare in-hospital safety outcomes in octogenarians and nonagenarians vs. younger patients. The primary outcome was a composite of in-hospital all-cause mortality or stroke. Secondary outcomes included procedural complications, length of stay (LOS), and total costs. Outcomes were determined using logistic regression models. Among 84 140 patients hospitalized for LAAO, 32.9% were octogenarians, 2.8% were nonagenarians, and 64.3% were ≤79 years of age. Over the study period, the volume of LAAO increased in all age groups (all *P*_trend_ < 0.01). After adjustment for clinical and demographic factors, octogenarians and nonagenarians had similar odds of in-hospital all-cause mortality or stroke [adjusted odds ratio (aOR) 1.41, 95% confidence interval (CI) 0.93–2.13 for octogenarians; aOR 1.69, 95% CI 0.67–3.92 for nonagenarians], cardiac tamponade, acute kidney injury, major bleeding, and blood transfusion, in addition to similar LOS and total costs compared with younger patients (all *P* > 0.05). However, octogenarians and nonagenarians had higher odds of vascular complications compared with younger patients (aOR 1.47, 95% CI 1.08–1.99 for octogenarians; aOR 1.60, 95% CI 1.18–2.97 for nonagenarians).

**Conclusion:**

Octogenarians and nonagenarians undergoing LAAO have a similar safety profile compared with clinically similar younger patients except for higher odds of vascular complications.

## Graphical Abstract

**Graphical Abstract:**
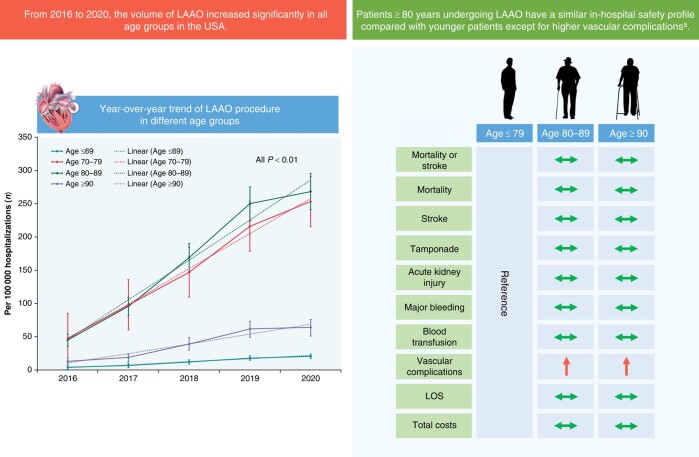
Summary of trends and in-hospital outcomes of LAAO in octogenarians (80–89 years) and nonagenarians (≥90 years) vs. younger patients (≤79 years). ^a^The multivariable regression model is adjusted for sex, race, insurance, income, hospital location and teaching status, bed size, region, type of admission, Elixhauser and Charlson comorbidity index scores, and relevant comorbidities. LAAO, left atrial appendage occlusion; LOS, length of stay.

**What’s new?**

- From 2016 to 2020, the volume of left atrial appendage occlusion (LAAO) increased significantly in all age groups in the USA.
- Compared with younger patients, octogenarians and nonagenarians undergoing LAAO had similar adjusted odds of in-hospital mortality and most procedural complications but higher adjusted odds of vascular complications.
- Length of stay and total costs of LAAO hospitalizations were similar between octogenarians and nonagenarians versus younger patients.
- Age alone should not preclude LAAO in otherwise suitable candidates.

## Introduction

Atrial fibrillation (AF) affects up to one in three individuals in the USA during their lifetimes and causes profound morbidity and mortality.^1^ Atrial fibrillation increases the risk of cardioembolic stroke, with the left atrial appendage being the most common site for thrombus formation.^2,3^ Stroke prevention remains the cornerstone of AF management, with oral anticoagulation (OAC) as the main therapy to reduce the risk of thromboembolism.^4–7^ According to a recent European Heart Rhythm Association consensus statement, OAC therapy is beneficial in all AF patients with non-sex-related CHA_2_DS_2_-VASc stroke risk factor(s), irrespective of their frailty status.^4^ Left atrial appendage occlusion (LAAO) provides a viable alternative to OAC in individuals with non-valvular AF with contraindications for long-term OAC, supported by multiple European and US professional society recommendations.^4–7^

Based on the most recent US census, the elderly population, encompassing octogenarians (80–89 years) and nonagenarians (≥90 years), continues to increase significantly, growing at a rate nearly five times that of the general population and representing a significant portion of patients with non-valvular AF.^8,9^ Both AF prevalence (6.4% at 65–69 years to 28.5% at 85 years) and incidence (14.2 per 1000 person-years at 65–69 years to 50.8 per 1000 person-years at 85 years) increase with age.^10^ Given their increased risks for major bleeding due to falls and medical comorbidities, elderly patients especially may benefit from LAAO as an alternative to OAC.^9^ Among the prior studies on LAAO, evaluation of outcomes among octogenarians and nonagenarians has been limited. Therefore, we analysed the National Inpatient Sample (NIS) database to evaluate the outcomes of LAAO in octogenarians and nonagenarians vs. younger patients.

## Methods

### Data source and ethics statement

Hospitalization data were abstracted from the NIS database, which is part of the Healthcare Cost and Utilization Project (HCUP) family of databases sponsored by the Agency for Healthcare Research and Quality.^11^ The NIS is the largest publicly available, fully deidentified, all-payer inpatient healthcare database in the USA. The NIS is derived from billing data submitted by hospitals to statewide organizations across the USA and has reliable and verified patient linkage numbers that can be used to track patients across hospitals within each state while adhering to strict privacy guidelines. The NIS database contains both patient- and hospital-level information from ∼1000 hospitals and represents ∼20% of all US hospitalizations, covering >7 million unweighted hospitalizations each year. When weighted, the NIS extrapolates to the national level, representing 35 million hospitalizations each year. Up to 40 discharge diagnoses and 25 procedure codes are collected for each patient using the *International Classification of Diseases, Tenth Revision* (*ICD-10*), codes.^12^ The NIS is compiled annually, which allows for the analysis of procedural trends over time.^13^ This study was exempt from the requirements of the Mayo Clinic Institutional Review Board because the NIS is a publicly available database comprised of deidentified data.

### Study population and patient selection

**Figure 1:**
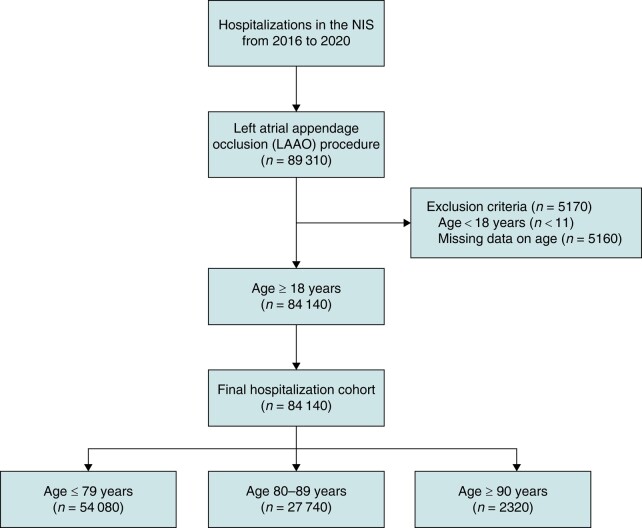
Study flow diagram showing inclusion and exclusion criteria. Hospitalization counts represent national-level estimates. NIS, National Inpatient Sample.

We queried the NIS database from January 2016 to December 2020 to identify hospitalizations in which adult patients underwent percutaneous LAAO (*ICD-10*, Procedure Coding System 02L73DK in any procedural field). We excluded hospitalizations with missing data on age and hospitalizations for patients <18 years of age. For hospitalizations that met inclusion criteria, we then stratified the patients into three cohorts based on age: octogenarians (80–89 years), nonagenarians (≥90 years), and patients ≤79 years of age (*Figure 1*). All ICD-10 diagnosis and procedure codes used in this study can be found in Supplementary material online, *Table S1*.

The NIS combines ‘race’ and ‘ethnicity’ into one data element (race). If both ‘race’ and ‘ethnicity’ were available, HCUP preferred ethnicity over race in assigning a value for the ‘race’ variable.^14^ Similar to prior NIS studies, three racial/ethnic groups with small sample sizes (Asian or Pacific Islander, Native American, and other) were combined into a single ‘other’ group to facilitate the analysis.^15–17^ The other three HCUP racial/ethnic groups (White, Black, and Hispanic) were left unchanged for the study. The ‘White’ group refers to non-Hispanic White patients, the ‘Black’ group refers to non-Hispanic Black patients, and the ‘Hispanic’ group refers to Hispanic patients of all races and origins.

### Study outcomes

The temporal trends in LAAO in different age groups were reported. The primary outcome was a composite of in-hospital all-cause mortality or stroke. Secondary outcomes were evaluated individually and as a composite of combined outcomes and included in-hospital all-cause mortality, stroke, cardiac tamponade, acute kidney injury (AKI), major bleeding, need for blood transfusion, and vascular complications (defined as a composite of arteriovenous fistula, aneurysm, haematoma, retroperitoneal bleeding, or venous thromboembolism). We also evaluated hospital length of stay (LOS), total hospital costs (inflation-adjusted to 2020 US dollars^18^), and discharge disposition. Charge-to-cost ratio files were used to convert charges to costs at the individual hospital level. Additional analysis comparing in-hospital outcomes of LAAO in nonagenarians vs. octogenarians was also performed.

### Statistical analysis

Descriptive statistics were presented as frequencies with percentages for categorical variables and as medians with interquartile ranges (IQRs) for continuous variables. Categorical variables were compared using the Pearson χ^2^ test or Fisher exact test as appropriate. Continuous variables were compared using the Kruskal–Wallis one-way ANOVA.

Temporal changes in hospitalizations for LAAO were analysed using linear regression analysis. A multivariable logistic regression analysis was used to compare in-hospital outcomes of LAAO between octogenarians and nonagenarians vs. younger patients. The model included the following variables: sex, race, insurance, income by ZIP code, hospital location and teaching status, bed size, region, type of admission (elective/non-elective and weekend/weekday), Elixhauser and Charlson comorbidity index scores, and specific relevant comorbidities (see Supplementary material online, *Table S2*). Adjustment variables were selected *a priori* on the basis of their clinical significance, which may directly influence in-hospital outcomes. The results from these models are presented as adjusted odds ratios (aORs) with 95% confidence intervals (CIs).

A complete set of data was available for all variables except for biological sex (<0.1%), race/ethnicity (3.2%), insurance (1.4%), and median household income (1.4%). As the overall missing values were minimal (<3.5%), they were assumed to be missing at random. Missing values were handled with listwise deletion and were not included in the logistic regression analysis.

In accordance with the HCUP data use agreement, we did not report variables that contained a small number of observed (i.e. unweighted) hospitalizations (<11) as this could pose risks of subject identification and data privacy violation.^19^ A two-tailed *P* < 0.05 was considered statistically significant. All statistical analyses were performed using Stata version 17 (StataCorp, College Station, TX) software, accounting for the NIS sampling design, and were weighted using sampling weights provided with the NIS database to estimate national-level effects per HCUP-NIS recommendations.^13^

## Results

### Patient and hospital characteristics

From 2016 to 2020, an estimated 84 140 hospitalized patients met inclusion criteria including an estimated 32.9% octogenarians, 2.8% nonagenarians, and 64.3% ≤79 years of age (*Figure 1*).

Octogenarians and nonagenarians were more likely to be female and White with Medicare insurance compared with younger patients (all *P* < 0.01). Octogenarians and nonagenarians had lower Elixhauser and Charlson comorbidity index scores compared with younger patients (both *P* < 0.01). This difference in comorbidities was driven mainly by the lower rates of diabetes mellitus, nicotine/tobacco use, alcohol and drug abuse, obesity, liver disease, chronic pulmonary disease, obstructive sleep apnoea, and depression in octogenarians and nonagenarians (all *P* < 0.01). However, octogenarians were more likely to have coronary artery disease and nonagenarians were more likely to have renal failure and congestive heart failure compared with younger patients (all *P* < 0.01). Differences were also found in the socioeconomic makeup of patients in each age group, with 26.0% of octogenarians and 33.0% of nonagenarians living in the highest median household income neighbourhood quartile compared with 22.6% of younger patients (*P* < 0.01). Baseline characteristics stratified by age groups are shown in *Table 1*.

**Table 1:** Baseline characteristics stratified by age groups

	Age groups			
	≤79 years (*n* = 54 080)	80–89 years (*n* = 27 740)	≥90 years (*n* = 2320)	*P*
**Demographic characteristics**				
Age	72 (68–76)	83 (81–85)	92 (90–94)	<0.01
Biological sex				
Male	32 410 (59.9)	15 425 (55.6)	1260 (54.4)	<0.01
Female	21 670 (40.1)	12 315 (44.4)	1055 (45.6)	
Race/ethnicity				
White	45 100 (86.2)	24 135 (89.8)	2050 (91.3)	<0.01
Black	2905 (5.6)	660 (2.5)	40 (1.8)	
Hispanic	2485 (4.7)	1220 (4.5)	105 (4.7)	
Other	1840 (3.5)	865 (3.2)	50 (2.2)	
Insurance				
Medicare	45 655 (86.2)	26 330 (95.8)	2195 (94.7)	<0.01
Medicaid	1005 (1.9)	90 (0.3)	<11 (<0.5)^a^	
Private insurance	6060 (11.4)	965 (3.6)	105 (4.5)	
Self-pay	245 (0.5)	95 (0.3)	<11 (<0.5)^a^	
Income quartile^b^				
I	12 410 (23.3)	5440 (19.8)	315 (13.8)	<0.01
II	14 335 (26.9)	7145 (26.0)	615 (27.0)	
III	14 475 (27.2)	7720 (28.1)	595 (26.2)	
IV	12 040 (22.6)	7135 (26.1)	750 (33.0)	
**Hospital characteristics**				
Location/teaching status				
Rural	1355 (2.5)	500 (1.8)	35 (1.5)	0.01
Urban non-teaching	5525 (10.2)	2750 (9.9)	300 (12.9)	
Urban teaching	47 200 (87.3)	24 490 (88.3)	1985 (85.6)	
Bed size^c^				
Small	5535 (10.2)	3120 (11.2)	240 (10.3)	0.12
Medium	12 600 (23.3)	6790 (24.5)	560 (24.2)	
Large	35 945 (66.5)	17 830 (64.3)	1520 (65.5)	
Region				
Northeast	8530 (15.8)	4660 (16.8)	465 (20.0)	<0.01
Midwest	12 570 (23.2)	5975 (21.5)	495 (21.3)	
South	22 125 (40.9)	10 585 (38.2)	735 (31.7)	
West	10 855 (20.1)	6520 (23.5)	625 (27.0)	
Elective admission	49 715 (92.1)	25 620 (92.5)	2110 (91.1)	0.50
Weekend admission	455 (0.8)	250 (0.9)	25 (1.1)	0.78
**Clinical characteristics**				
Elixhauser comorbidity index	4 (3–6)	4 (3–5)	4 (3–5)	<0.01
Charlson comorbidity index	2 (1–4)	2 (1–3)	2 (1–3)	<0.01
0	11 175 (20.7)	6080 (21.9)	575 (24.7)	0.06
1	13 040 (24.0)	6655 (24.0)	550 (23.7)	
2	10 315 (19.1)	5530 (19.9)	405 (17.5)	
≥3	19 550 (36.2)	9475 (34.2)	790 (34.1)	
Diabetes mellitus	21 400 (39.6)	8145 (29.4)	400 (17.2)	<0.01
Hypertension	47 230 (87.3)	24 005 (86.5)	1975 (85.1)	0.16
Dyslipidaemia	33 685 (62.3)	17 410 (62.8)	1455 (62.7)	0.83
Nicotine/tobacco use	21 835 (40.4)	10 115 (36.5)	730 (31.5)	<0.01
Alcohol abuse	1055 (2.0)	165 (0.6)	15 (0.6)	<0.01
Drug abuse	245 (0.5)	45 (0.2)	<11 (<0.5)^a^	<0.01
Obesity	11 875 (22.0)	2955 (10.7)	75 (3.2)	<0.01
Coronary artery disease	26 160 (48.4)	14 410 (51.9)	1120 (48.3)	<0.01
Peripheral vascular disease	8580 (15.9)	4660 (16.8)	335 (14.4)	0.19
Congestive heart failure	21 385 (39.5)	11 135 (40.1)	1105 (47.6)	<0.01
Renal failure	12 660 (23.4)	7015 (25.3)	735 (31.7)	<0.01
Dialysis dependent	1940 (3.6)	385 (1.4)	15 (0.6)	<0.01
Liver disease	1945 (3.6)	400 (1.4)	<11 (<0.5)^a^	<0.01
Chronic pulmonary disease	12 815 (23.7)	5715 (20.6)	350 (15.1)	<0.01
Obstructive sleep apnoea	11 710 (21.7)	3565 (12.9)	145 (6.3)	<0.01
Coagulopathy	2265 (4.2)	1055 (3.8)	120 (5.2)	0.25
Cancer	1150 (2.1)	665 (2.4)	55 (2.4)	0.52
Malnutrition	85 (0.2)	50 (0.2)	<11 (<0.5)^a^	0.37
Dementia	970 (1.8)	1300 (4.7)	100 (4.3)	<0.01
Depression	4955 (9.2)	1750 (6.3)	105 (4.5)	<0.01
Previous history				
Myocardial infarction	7420 (13.7)	3380 (12.2)	235 (10.1)	<0.01
Stroke/TIA	12 250 (22.7)	6830 (24.6)	545 (23.5)	0.01
Cardiac arrest	360 (0.7)	100 (0.4)	<11 (<0.5)^a^	0.01
PCI	9110 (16.8)	4795 (17.3)	450 (19.4)	0.32
CABG	6980 (12.9)	4290 (15.5)	315 (13.6)	<0.01
ICD	3775 (7.0)	1660 (6.0)	105 (4.5)	<0.01
PPM	7735 (14.3)	6510 (23.5)	710 (30.6)	<0.01

Data are presented as median (IQR) or *n* (%). Two authors (M.I. and H.A.) independently verified the *International Classification of Diseases, Tenth Revision* (*ICD-10*), codes that corresponded to each comorbidity (see Supplementary material online, *Table S1*), and any disagreements in inclusion or exclusion of *ICD-10* codes were discussed with a third author (M.A).

CABG, coronary artery bypass grafting; ICD, implantable cardioverter-defibrillator; IQR, interquartile range; PCI, percutaneous coronary intervention; PPM, permanent pacemaker; TIA, transient ischaemic attack.

^a^Cell counts < 11 are not reportable per HCUP guidelines.

^b^Estimated median household incomes are ZIP code-specific, updated annually, and classified into four quartiles indicating the poorest to wealthiest populations.

^c^Bed size categories are based on inpatient beds and are specific to the hospital’s location and teaching status. A more detailed explanation of all the variables in the NIS, including the specific dollar amounts in each category of median household income and the number of hospital beds in each category, is available online (https://www.hcup-us.ahrq.gov/db/nation/nis/nisdde.jsp).

### Temporal trends

**Figure 2:**
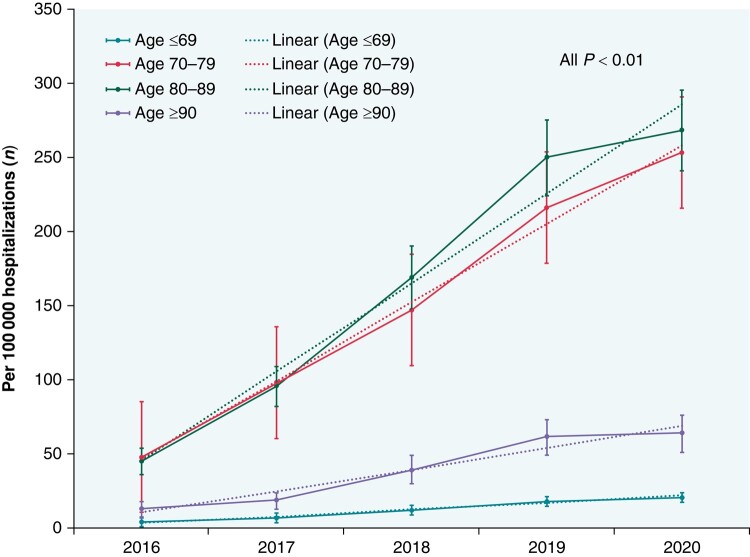
Year-over-year trend of LAAO procedure in septuagenarians, octogenarians, nonagenarians, and patients aged ≤69 years. Error bars represent 95% confidence intervals. LAAO, left atrial appendage occlusion.

From 2016 to 2020, the number of LAAO procedures per 100 000 hospitalizations increased significantly from 48 to 253 in septuagenarians, 45 to 268 in octogenarians, 13 to 64 in nonagenarians, and 4 to 21 in patients aged ≤69 years (all *P*_trend_ < 0.01). Annual trends for LAAO procedure in different age groups are shown in *Figure 2*.

### In-hospital outcomes

The estimated overall rate of in-hospital all-cause mortality or stroke among patients undergoing LAAO was 0.9% (95% CI 0.7–1.0%). After adjustment for potential confounders using multivariable regression analysis, octogenarians and nonagenarians had similar odds of the primary composite outcome of in-hospital all-cause mortality or stroke and similar odds of individual secondary outcomes including in-hospital mortality, stroke, cardiac tamponade, AKI, major bleeding, and blood transfusion as well as combined secondary outcomes compared with younger patients (all *P* > 0.05). The odds of vascular complications were higher in octogenarians and nonagenarians compared with younger patients (aOR 1.47, 95% CI 1.08–1.99 for octogenarians; aOR 1.60, 95% CI 1.18–2.97 for nonagenarians).

**Figure 3:**
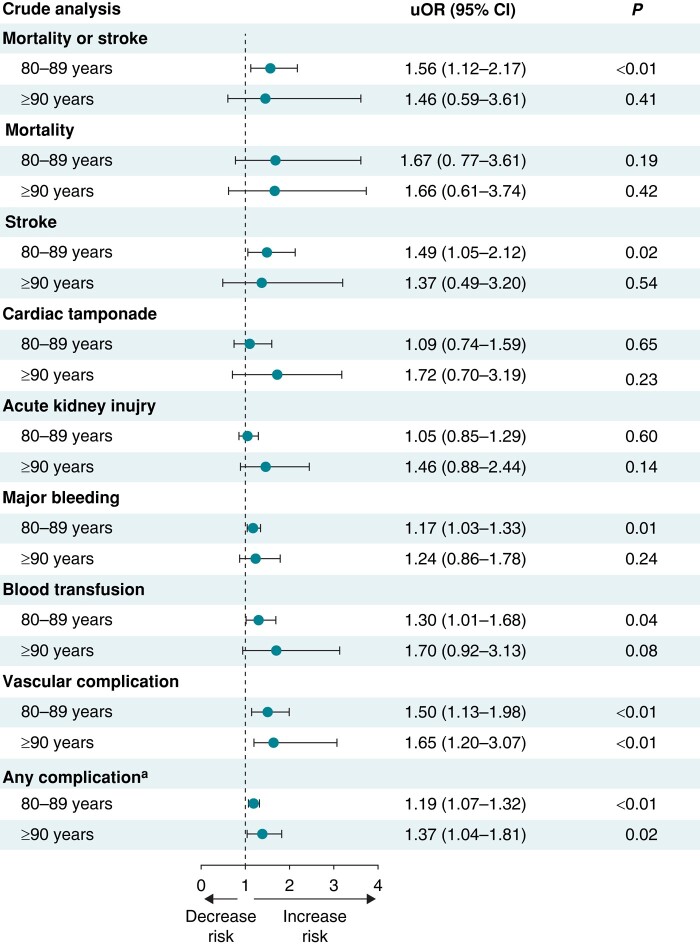
Forest plot showing crude analysis for LAAO outcomes in octogenarians (80–89 years) and nonagenarians (≥90 years) vs. younger patients (≤79 years; reference). ^a^Defined as a composite of in-hospital mortality, stroke, cardiac tamponade, acute kidney injury, major bleeding, blood transfusion, or vascular complication. LAAO, left atrial appendage occlusion; uOR, unadjusted odds ratio; CI, confidence interval.

**Figure 4:**
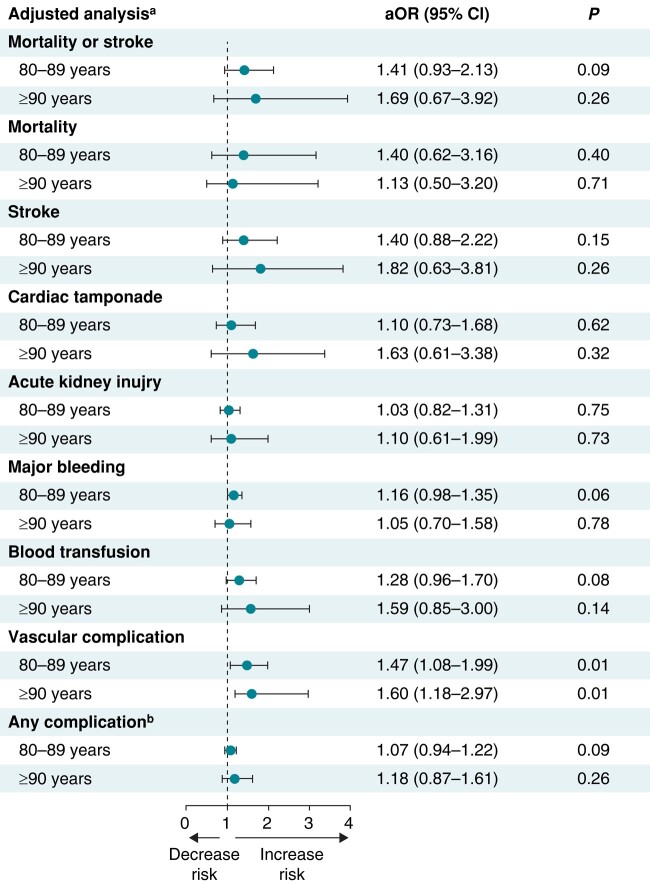
Forest plot showing adjusted analysis for LAAO outcomes in octogenarians (80–89 years) and nonagenarians (≥90 years) vs. younger patients (≤79 years; reference). ^a^Adjusted analysis based on sex, race, insurance, income, hospital location and teaching status, bed size, region, type of admission, Elixhauser and Charlson comorbidity index scores, and relevant comorbidities. ^b^Defined as a composite of in-hospital mortality, stroke, cardiac tamponade, acute kidney injury, major bleeding, blood transfusion, or vascular complication. LAAO, left atrial appendage occlusion; aOR, adjusted odds ratio; CI, confidence interval.

Octogenarians and nonagenarians had a similar median LOS of 1 day (*P* = 0.40) and similar median total costs ($28 911 and $30 255 vs. $28 988, respectively, *P* = 0.10) compared with younger patients. For hospitalizations in which the patient was discharged alive, octogenarians and nonagenarians were discharged at greater rates to a skilled nursing facility or home healthcare as opposed to home. In-hospital outcomes stratified by age groups are shown in *Table 2* and in *Figures 3* and *4*.

**Table 2:** Unadjusted in-hospital outcomes stratified by age groups

	Age groups			
	≤79 years (*n* = 54 080)	80–89 years (*n* = 27 740)	≥90 years (*n* = 2320)	*P*
**Clinical outcomes**				
Mortality or stroke	400 (0.7)	320 (1.2)	25 (1.1)	0.02
Mortality	70 (0.1)	60 (0.2)	<11 (<0.5)^a^	0.40
Stroke	340 (0.6)	260 (0.9)	20 (0.9)	0.08
Cardiac tamponade	340 (0.6)	190 (0.7)	25 (1.1)	0.45
Acute kidney injury	1285 (2.4)	695 (2.5)	80 (3.4)	0.31
Major bleeding	3140 (5.8)	1875 (6.8)	165 (7.1)	0.03
Blood transfusion	760 (1.4)	505 (1.8)	55 (2.4)	0.04
Vascular complications	535 (1.0)	410 (1.5)	37 (1.6)	0.01
Any complication^b^	5005 (9.3)	3015 (10.9)	285 (12.3)	<0.01
**Discharge disposition**				
Routine	50 840 (94.1)	25 170 (90.7)	1995 (86.0)	<0.01
Transfer to short-term hospital	30 (0.1)	35 (0.1)	<11 (<0.5)^a^	
Transfer to SNF or ICF	1100 (2.0)	755 (2.7)	105 (4.5)	
Home healthcare	1985 (3.7)	1715 (6.2)	215 (9.3)	
**Resource utilization**				
LOS (days)	1 (1–1)	1 (1–1)	1 (1–1)	0.40
Hospital cost ($)	28 988 (20 620–42 283)	28 911 (21 120–43 507)	30 255 (21 655–43 491)	0.10

Data are presented as median (IQR) or *n* (%). The *International Classification of Diseases, Tenth Revision* (*ICD-10*), codes corresponding to each of the in-hospital outcomes were identified with the same process used to identify comorbidity codes (see Supplementary material online, *Table S1*).

ICF, intermediate care facility; IQR, interquartile range; LOS, length of stay; SNF, skilled nursing facility.

^a^Cell counts < 11 are not reportable per HCUP guidelines.

^b^Defined as a composite of in-hospital mortality, stroke, cardiac tamponade, acute kidney injury, major bleeding, blood transfusion, or vascular complications.

In an additional analysis comparing in-hospital outcomes of LAAO in nonagenarians vs. octogenarians, there were no significant differences in the adjusted odds of the primary composite outcome of in-hospital all-cause mortality or stroke, as well as individual and combined secondary outcomes including in-hospital mortality, stroke, cardiac tamponade, AKI, major bleeding, blood transfusion, and vascular complications (all *P* > 0.05) (see Supplementary material online, *Figure S1*).

## Discussion

This large national database study demonstrates three principal findings (Graphical Abstract): (i) LAAO procedure rates increased significantly in all age groups across the 5-year study period; (ii) compared with younger patients, octogenarians and nonagenarians undergoing LAAO had similar adjusted odds of in-hospital mortality and most procedural complications but higher adjusted odds of vascular complications; and (iii) LOS and total costs were similar between all 3 age groups.

### Trends in left atrial appendage occlusion procedure

Across the 5-year period, our study found that LAAO rates increased significantly among all age groups. The increased prevalence of LAAO noted during the 5-year period parallels a prior study by Shrestha *et al.*^20^ who found a significant increase in annual WATCHMAN device implantation from 2016 to 2019 (*P* = 0.01). Increased use of LAAO is likely multifactorial in aetiology, with increased experience and number of LAAO operators and decreasing rates of post-procedural complications as contributing factors.^20^ A study analysing adverse procedural outcomes after LAAO by Khalil *et al.*^21^ highlighted the improving safety outcomes of WATCHMAN implantation, noting a decreased incidence of post-procedural complications compared with prior studies, driven by increased operator skill and maturity of the procedure. The increased popularity of LAAO among octogenarians and nonagenarians is also likely due to the overall appeal and feasibility of LAAO, serving as a favourable alternative in mitigating the thromboembolic risk in elderly individuals with non-valvular AF who are unable to tolerate anticoagulation.^22–24^ The cost-effectiveness of LAAO compared with long-term use of anticoagulation, as demonstrated in prior studies, also likely contributed to the increased prevalence of LAAO among all age groups.^24^

### In-hospital outcomes

Octogenarians and nonagenarians undergoing LAAO in our study had similar odds of in-hospital mortality and most procedural complications compared with younger patients. This is congruent with prior studies showing no significant difference in procedural complications following AF catheter ablation among patients ≥75 years old compared with younger patients (65–74 years old).^25^ In addition, in a recent study by Alkhouli *et al.*, the average treatment effects of LAAO (compared with warfarin) were similar in the elderly population (compared with younger patients).^26^ These findings of similar LAAO safety outcomes and treatment effects^26^ have significant implications for evaluating the candidacy of patients based on age because; although elderly patients may be frailer, these findings suggest that they should not be excluded from undergoing LAAO based upon age alone if they are otherwise eligible for the procedure. However, certain comorbidities such as advanced chronic kidney disease (CKD), heart failure, and cirrhosis, and a higher CHA_2_DS_2_-VASc score have been shown to influence clinical outcomes of LAAO.^27–30^ In a study evaluating the prognostic value of CKD in patients undergoing LAAO, moderate-to-severe CKD was associated with a higher incidence of the primary composite endpoint of cardiovascular mortality, thromboembolism, and major bleeding.^27^ Similarly, among patients undergoing LAAO, the presence of heart failure was associated with prolonged LOS and higher hospitalization costs,^28^ the presence of cirrhosis was associated with higher rates of 30-day readmission,^29^ and the presence of a higher CHA_2_DS_2_-VASc score was associated with an increased risk of peri-procedural complications and resource utilization.^30^ Female sex has also been associated with worse in-hospital outcomes following LAAO compared with men.^31^

The higher odds of vascular complications in octogenarians and nonagenarians undergoing LAAO compared with younger patients may be attributed in part to the increased likelihood of octogenarians and nonagenarians being female, as prior studies found that women are more susceptible to developing vascular complications after procedural interventions owing to their complex vascular anatomy and smaller body size.^32^ Further studies are needed to evaluate the long-term outcomes of LAAO in elderly patients after discharge.

### Length of stay and hospital cost

Our study found similar LOS and total costs between all three age groups hospitalized for LAAO. These findings align with prior studies, which found that elderly age (>80 years) was not associated with increased hospitalization costs compared with younger patients who underwent LAAO.^22^ In another study analysing outcomes among elderly individuals who underwent LAAO, the difference in index admission costs between patients ≥80 and <80 years old was noted to be non-significant (*P* = 0.38).^33^ However, when stratified by those who experienced in-hospital adverse events to those who did not, individuals who experienced in-hospital adverse events of both age groups were found to have significantly higher hospitalization costs (*P* < 0.01), highlighting the significance of post-procedural LAAO complications on hospitalization costs.^33^ Similarly, Piayda *et al.*^34^ found that procedural and device-related adverse events occurring after LAAO were associated with a significantly prolonged duration of hospitalization (*P* < 0.01). The similar LOS and total costs noted in our study are likely mostly attributable to the relatively modest variation in in-hospital mortality and most procedural complications, resulting in similar hospital courses among all three age groups.

### Limitations

Our study has several important limitations. First, in a retrospective NIS study using administrative claims codes, incorrect coding could lead to inaccurate data. Second, the retrospective nature of the study makes it potentially subject to selection bias. Third, some outcomes and clinical characteristics were not available per the HCUP data use agreement because patient counts were <11. Fourth, the NIS does not provide data on the specific cause of in-hospital mortality. Fifth, we adjusted for a broad array of sociodemographic characteristics and medical comorbidities, but detailed baseline and procedural characteristics such as echocardiographic findings, LAAO access site, LAAO device type, intra-procedural imaging use, peri-procedural medications, and operator experience were unavailable, which may have led to unmeasured confounding. Finally, our outcomes were confined to in-hospital mortality and complications during the index hospitalization. Studies exploring the long-term outcomes of LAAO in elderly patients are still needed.

Despite these limitations, we believe that our study adds meaningfully to the literature by describing LAAO outcomes among octogenarians and nonagenarians with adjustment for demographic and clinical characteristics. The NIS is well validated for outcomes studies like ours and undergoes serial data accuracy checks and quality control. In addition, the NIS data are geographically diverse including a nationally representative sample of centres and operators and, hence, reliably demonstrate real-world practice and outcomes.

## Conclusions

Octogenarians and nonagenarians undergoing LAAO have a similar safety profile compared with clinically similar younger patients except for higher odds of vascular complications. Age alone should not preclude LAAO in otherwise suitable candidates.

## Supplementary material

Supplementary material is available at *Europace* online.

## Supplementary Material

euae055_Supplementary_Data

## Data Availability

The NIS data are publicly available for purchase online from the Agency for Healthcare Research and Quality.
