# Supplementary material for: In-hospital safety outcomes of left atrial appendage occlusion in octogenarians and nonagenarians
Source: Europace. 2024 Feb 23;26(3):euae055. doi: 10.1093/europace/euae055 (PMC10927254; doi:10.1093/europace/euae055)

**Supplementary Material**

**Table S1.** ICD-10 diagnosis (CM) and procedure (PCS) codes

| **Procedures** | **ICD-10-PCS codes** |
| --- | --- |
| Percutaneous LAAO | 02L73DK |

| **Baseline characteristics** | **ICD-10 CM codes** |
| --- | --- |
| **Comorbidities** | |
| Diabetes mellitus | E10.0, E10.1, E10.9, E11.0, E11.1, E11.9, E12.0, E12.1, E12.9, E13.0, E13.1, E13.9, E14.0, E14.1, E14.9, E10.2-E10.8, E11.2-E11.8, E12.2-E12.8, E13.2-E13.8, E14.2-E14.8 |
| Hypertension | I10.x, I11.x-I13.x, I15.x |
| Dyslipidemia | E78.x |
| Nicotine/tobacco use | F17.x, Z72.0, Z87.891 |
| Alcohol abuse | F10, E52, G62.1, I42.6, K29.2, K70.0, K70.3, K70.9, T51.x, Z50.2, Z71.4, Z72.1 |
| Drug abuse | F11.x-F16.x, F18.x, F19.x,  Z71.5. Z72.2 |
| Obesity | E66.x |
| Coronary artery disease | I25.x |
| Peripheral vascular disease | I70.x, I71.x, I73.1, I73.8, I73.9, I77.1, I79.0, I79.2, K55.1, K55.8, K55.9, Z95.8, Z95.9 |
| Congestive heart failure | I09.9, I11.0, I13.0, I13.2, I25.5, I42.0, 142.5-I42.9, I43.x, I50.x, P29.0 |
| Renal failure | I12.0, I13.1, N18.x, N19.x, N25.0, Z49.0-Z49.2, Z94.0, Z199.2 |
| Dialysis dependent | Z99.2 |
| Liver disease | B18.x, I85.x, I86.4, I98.2, K70.x, K71.1, K71.3-K71.5, K71.7, K72.x-K74.x, K76.0, K76.2-K76.9. Z94.4 |
| Chronic pulmonary disease | I27.8, 127.9, J40.x-J47.x, J60.x-J67.x, J68.4, J70.1, J70.3 |
| Obstructive sleep apnea | G47.33 |
| Coagulopathy | D65-D68.x, D69.1, D69.3-D69.6 |
| Cancer | C0x.x, C1x.x, C2x.x, C30.x, C31.x, C32.x, C33.x, C34.x, C37.x, C38.x, C39.x, C40.x, C41.x, C43.x, C45.x, C46.x, C47.x, C48.x, C49.x, C50, C51-58.x, C60-63.x, C76.x, C80.1, C81.x, C82.x, C83.x, C84.x, C85.x, C88.x, C9x.x |
| Malnutrition | E43, E44.x, E45, E46 |
| Dementia | F01.x, F02.x, F03.x, F04, F05, F06.1, F06.8, G13.2, G13.8, G30.x, G31.0x, G31.1, G31.2, G91.4, G94, R41.81, R54 |
| Depression | F20.4, F31.3-F31.5, F32.x, F33.x, F34.1, F41.2, F43.2 |
| **Previous history** | |
| Myocardial infarction | I25.2 |
| Stroke/TIA | Z86.73 |
| Cardiac arrest | Z86.74 |
| PCI | Z98.61, Z95.5 |
| CABG | Z95.1 |
| ICD | Z95.810 |
| PPM | Z95.0 |

| **In-hospital outcomes** | **ICD-10 CM/PCS codes** |
| --- | --- |
| Stroke | I63.x, I67.81, I67.82, G45.x, G46.x, H34.0x, H34.1x, H34.2x, I60.x, I61.x, I62.x, I97.820, I97.810 |
| Cardiac tamponade | I31.4 |
| Acute kidney injury | N17.x, N99.0 |
| Major bleeding | I97.610, I97.410, I97.618, I97.418, L76.22, L76.02, I97.51, L76.12, K92.0, K92.1, K92.2, K91.841, K91.62, R31.0, N99.821, N99.62, R04.x, J95.831, J95.62, R58, D62 |
| Need for blood transfusion | [30233H0](https://www.icd10data.com/ICD10PCS/Codes/3/0/2/3/30233H0), [30233H1](https://www.icd10data.com/ICD10PCS/Codes/3/0/2/3/30233H1), 30233N0, 30233N1, 30233P0, 30233P1, 30230H0, 30230H1, 30230N0, 30230N1, 30230P0, 30230P1, 30243H0, 30243H1, 30243N0, 30243N1, 30243P0, 30243P1, 30240H0, 30240H1, 30240N0, 30240N1, 30240P0, 30240P1 |
| Vascular complications | I77.0, I72.x, L76.32, L76.02, I97.630, I97.410, I97.638, I97.418, K66.1, I26.92, I26.93, I26.94, I26.99, I26.02, I26.09, I82.4x, T81.718A, T81.719A, T81.72XA |

**Table S2.** Variables used in the multivariable regression analysis to compute adjusted odds of in-hospital outcomes

| **Demographic characteristics** |
| --- |
| Biological sex |
| Race/ethnicity |
| Insurance |
| Income quartile |
| **Hospital characteristics** |
| Location/teaching status |
| Bed size |
| Region |
| Elective admission |
| Weekend admission |
| **Clinical characteristics** |
| Elixhauser comorbidity index |
| Charlson comorbidity index |
| Diabetes mellitus |
| Hypertension |
| Dyslipidemia |
| Nicotine/tobacco use |
| Alcohol abuse |
| Drug abuse |
| Obesity |
| Coronary artery disease |
| Peripheral vascular disease |
| Congestive heart failure |
| Renal failure |
| Dialysis dependent |
| Liver disease |
| Chronic pulmonary disease |
| Obstructive sleep apnea |
| Coagulopathy |
| Cancer |
| Malnutrition |
| Dementia |
| Depression |
| Previous history of myocardial infarction |
| Previous history of stroke/TIA |
| Previous history of cardiac arrest |
| Previous history of PCI |
| Previous history of CABG |
| Previous history of ICD |
| Previous history of PPM |

**Figure S1.** Forest plot showing crude and adjusted analysis for LAAO outcomes in nonagenarians (≥90 years) vs. octogenarians (80-89 years; Reference). ^a^Defined as a composite of in-hospital mortality, stroke, cardiac tamponade, acute kidney injury, major bleeding, blood transfusion, or vascular complication. ^b^Adjusted analysis based on sex, race, insurance, income, hospital location and teaching status, bed size, region, type of admission, Elixhauser and Charlson comorbidity index scores, and relevant comorbidities.


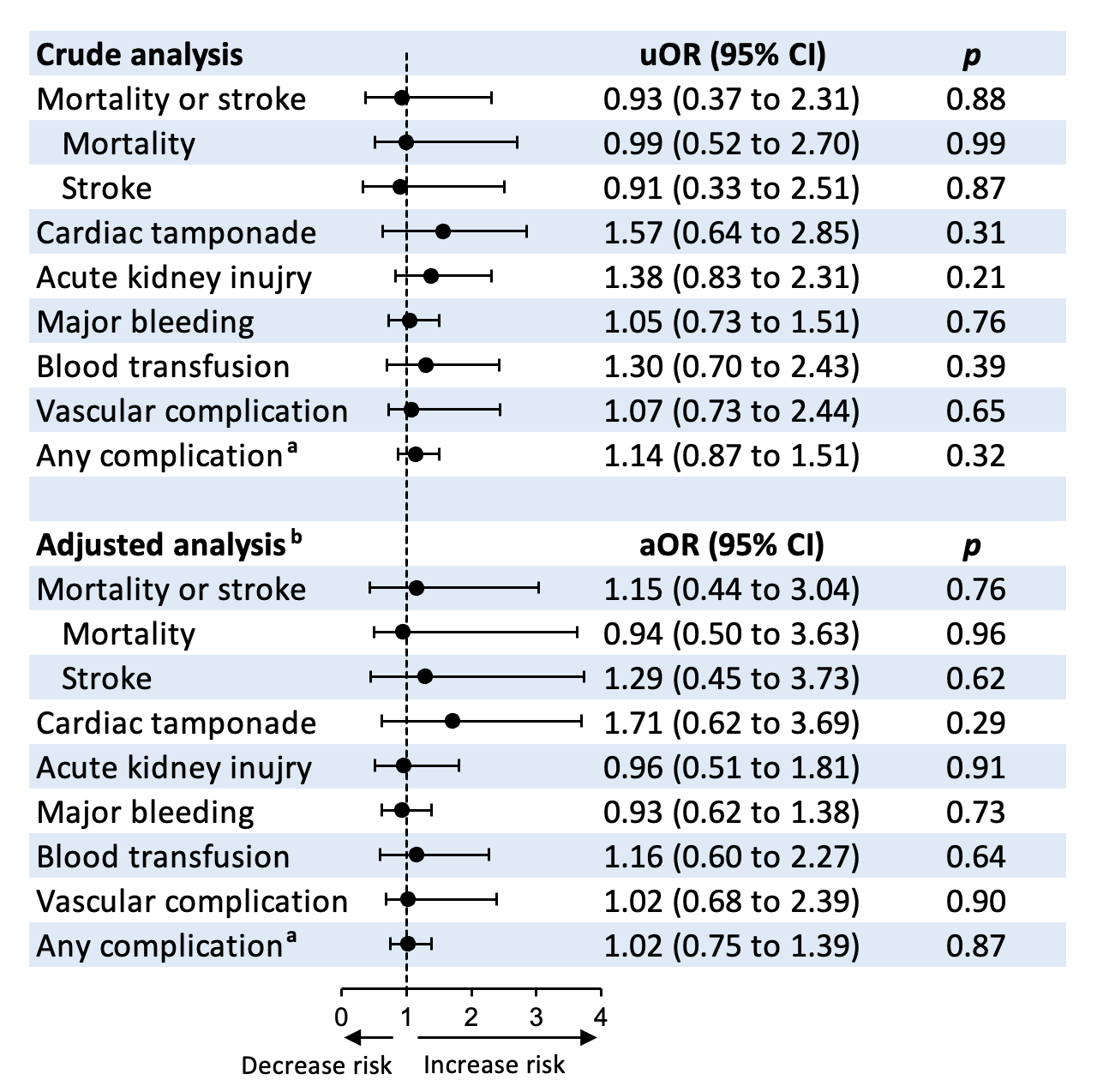

Supplement: euae055_Supplementary_Data [file euae055_supplementary_data.docx]
